# Nomogram Development for Predicting Synchronous Lung Metastasis in Patients with T1 Colorectal Cancer: An SEER-Based Analysis

**DOI:** 10.3390/medicina62030431

**Published:** 2026-02-25

**Authors:** Pin-Chun Chen, Yi-Kai Kao, Po-Wen Yang, Chia-Hung Chen, Chih-I Chen

**Affiliations:** 1 Division of Colon and Rectal Surgery, Department of Surgery, E-DA Hospital, I-Shou University, Kaohsiung 824, Taiwan; jamie3768@gmail.com (P.-C.C.); kevin.2512@hotmail.com (Y.-K.K.); cs5546570@gmail.com (P.-W.Y.); petechchen@gmail.com (C.-H.C.); 2 College of Management, National Sun Yat-sen University, Kaohsiung 804, Taiwan; 3 School of Medicine, I-Shou University, Kaohsiung 824, Taiwan

**Keywords:** colorectal cancer (CRC), synchronous lung metastasis (sLM), nomogram, SEER database

## Abstract

*Background and Objectives*: Colorectal cancer is a significant global health burden, with lung metastasis contributing substantially to mortality. Accurate risk stratification of synchronous lung metastasis (sLM) in patients with T1 colorectal cancer is important for informing staging decisions, yet no validated tool exists to guide selective chest computed tomography (CT) in this population. This study aimed to develop and validate two complementary nomograms: a clinicopathologic model (Model A) for pre-imaging risk stratification to guide chest CT decisions, and a post-staging model (Model B) incorporating concurrent organ metastasis status for comprehensive risk profiling. *Materials and Methods*: We utilized data from the Surveillance, Epidemiology, and End Results database, including patients diagnosed with T1 colorectal cancer between 2010 and 2020. Logistic regression analyses identified significant predictors of synchronous lung metastasis. A nomogram was constructed based on these predictors and validated using a split-sample approach. *Results*: The study included 41,728 patients with T1 colorectal cancer. Significant predictors of synchronous lung metastasis included tumor grade, size, location, lymph node involvement, and concurrent metastases in other organs. Two models were developed: Model A (clinicopathologic-only) demonstrated moderate discriminatory ability (AUC = 0.728, 95% CI: 0.710–0.746), while Model B (including concurrent organ metastasis status) demonstrated good discrimination (AUC = 0.856, 95% CI: 0.843–0.869): Model A validation AUC = 0.716; Model B validation AUC = 0.849. Calibration plots showed good agreement between predicted and observed probabilities of synchronous lung metastasis. *Conclusions*: This study developed and internally validated two nomograms for predicting sLM in patients with T1 CRC. Model A, using readily available clinicopathological factors, may support selective chest CT decisions during initial staging. Model B, incorporating post-staging information, may assist in prognostic counseling. External validation is required before clinical implementation.

## 1. Introduction

Colorectal cancer (CRC) is a significant global health burden and ranks as the third most common cancer and the second leading cause of cancer-related deaths worldwide [1,2]. The evolving epidemiology of CRC was further detailed by Siegel et al., 2023, who noted a disturbing shift towards diagnosis at a younger age and at a more advanced stage [3]. Metastasis significantly contributes to CRC-related mortality, with the lungs being one of the most common sites of distant metastasis. Approximately half of patients with CRC develop metastases during the disease course, with the liver being the most prevalent site, followed by the lungs and peritoneum [4,5,6]. The incidence of lung metastasis in patients with CRC varies across studies but generally falls within 5.2–11.0% [7,8,9]. Synchronous lung metastasis (sLM) in CRC is a significant clinical concern because of its association with poor prognosis and complex treatment strategies. Studies have shown that synchronous lung metastases occur in a notable subset of patients with CRC, with factors such as advanced T and N stages, elevated carcinoembryonic antigen (CEA) levels, and rectal cancer being strong predictors [10,11].

Early detection and accurate risk stratification of sLM in patients with CRC are crucial for improving prognosis and guiding treatment decisions. The incidence of sLM in patients with CRC is approximately 5.2%, with a median survival time of 10 months [7]. Several studies have identified key risk factors for sLM development, including older age, male sex, African American race, advanced T and N stages, poor histological differentiation, CEA positivity, and the presence of other metastases, such as liver, bone, and brain metastases [12,13,14]. Identifying high-risk patients can facilitate personalized surveillance strategies, targeted therapies, and timely interventions. Current National Comprehensive Cancer Network (NCCN) guidelines for stage I (T1N0) colorectal cancer do not uniformly mandate chest CT, and considerable institutional heterogeneity exists regarding the extent of metastatic workup in early-stage disease [15]. A validated risk stratification tool could support selective, evidence-based use of chest CT in this population, potentially avoiding unnecessary imaging in low-risk patients while ensuring timely detection in high-risk individuals. Our nomogram is designed to support this downstream clinical decision: it identifies which T1 CRC patients carry a meaningfully elevated lung metastasis risk and, therefore, warrant dedicated chest imaging. However, the identification of sLM in early-stage CRC, particularly T1 tumors, remains challenging because of the limited predictive tools available.

Recent advancements in predictive models, deep learning deployment, and biomarker identification have the potential to transform the current landscape of early-stage CRC metastasis detection and prognostication. To understand metastatic predictors in early-stage CRC, Mo et al. developed a novel predictive model for lymph node metastasis in patients with T1-2 CRC [16]. By incorporating seven significant predictors into a nomogram, they aimed to aid physicians in making informed treatment decisions, underscoring the significance of nuanced diagnostic tools in the management of early-stage disease. Echoing the need for advanced diagnostic strategies, Tang et al. [17] developed a nomogram based on risk and prognostic factors that demonstrated noteworthy performance. While their focus was on liver metastasis, the underlying theme of utilizing population-based studies to derive predictive tools is relevant to our goal of predicting lung metastasis in patients with T1 CRC.

This study aimed to develop and validate two complementary nomograms for predicting sLM in patients with T1 CRC: a clinicopathologic model (Model A) for pre-imaging risk stratification, and a full post-staging model (Model B) for comprehensive risk profiling. Using a large population-based dataset from the SEER database and incorporating key variables such as tumor location, grade, size, lymph node status, and concurrent organ metastasis status, nomograms can provide personalized risk assessment for individual patients at different stages of the diagnostic workup.

## 2. Materials and Methods

### 2.1. Data Source and Study Design

In this retrospective population-based study, patient data were obtained from the SEER database, an open public resource provided by the US National Cancer Institute. The SEER database is designed to provide information on cancer incidence, prevalence, survival, and mortality. SEER covers approximately 47.9% of the US population (17 registries, malignant only) and represents various demographic groups. The database collects a wide range of information, including patient demographics (age, sex, and race), tumor characteristics (site, stage, and morphology), treatment details, and follow-up data for survival analysis. Data from the SEER database are publicly accessible and can be downloaded for research purposes. The SEER*Stat version 8.4.3 (https://seer.cancer.gov/seerstat/) (accessed on 19 February 2025) (Information Management Service Inc., Calverton, MD, USA) was used in the present study [18].

### 2.2. Population

Data from patients aged > 18 years from 2010 to 2020 were extracted. The inclusion criterion was patients with T1 stage primary CRC at the time of entry. The exclusion criteria were as follows: unknown lung metastasis status, incomplete survival information, missing information on cancer-specific mortality, and missing covariates (race/ethnicity, tumor size, bone/brain/liver metastasis status, and N stage). The largest single source of exclusion was missing tumor size data (*n* = 19,422; 29.7% of eligible patients), which may introduce selection bias. A comparison of included versus excluded patients is provided in the main Results and Supplementary Table S3.

### 2.3. Data Collection and Statistical Analysis

Categorical data are presented as numbers (*n*) and percentages (%), and the chi-square test or Fisher’s exact test was used for comparison, as appropriate. Continuous data are expressed as mean ± standard deviation. Logistic regression models were used to estimate the odds ratio (OR) and 95% confidence interval (CI) for the risk of lung metastasis. Stepwise selection was used to identify the factors correlated with lung metastasis. The entry criteria were variables with a value of *p* < 0.5, and the remaining criteria were *p* < 0.05. All statistical analyses were conducted using the SAS software (version 9.4; SAS Institute Inc., Cary, NC, USA).

A nomogram was constructed based on the results of the multivariate analysis. Independent risk factors were used to construct nomograms for predicting lung metastasis. A training cohort was used to construct nomograms that were validated in the validation cohort. The area under the curve of the receiver operating characteristic (ROC) curves was calculated and calibration curves were plotted to assess the predictive power of the model. Analyses were performed using R software (version 4.2.2; R Foundation for Statistical Computing, Vienna, Austria). The following R packages were used: rms, ggplot2, rmda, pROC, and foreign. Statistical significance was defined as a two-tailed *p*-value < 0.05.

Two models were evaluated: Model A (clinicopathologic-only) included primary tumor site, tumor grade, tumor size, and N stage as predictors, and is intended for pre-imaging risk stratification to guide chest CT decisions. Model B (full post-staging model) included all Model A variables plus liver metastasis and bone metastasis status, and is intended for post-staging risk profiling when concurrent organ metastasis data are already available. This dual-model approach was adopted to address the concern that including concurrent organ metastasis variables as predictors of lung metastasis may inflate apparent discrimination through shared metastatic biology (predictor leakage), while still providing a clinically useful post-staging tool.

## 3. Results

### 3.1. Study Population

**Figure 1:**
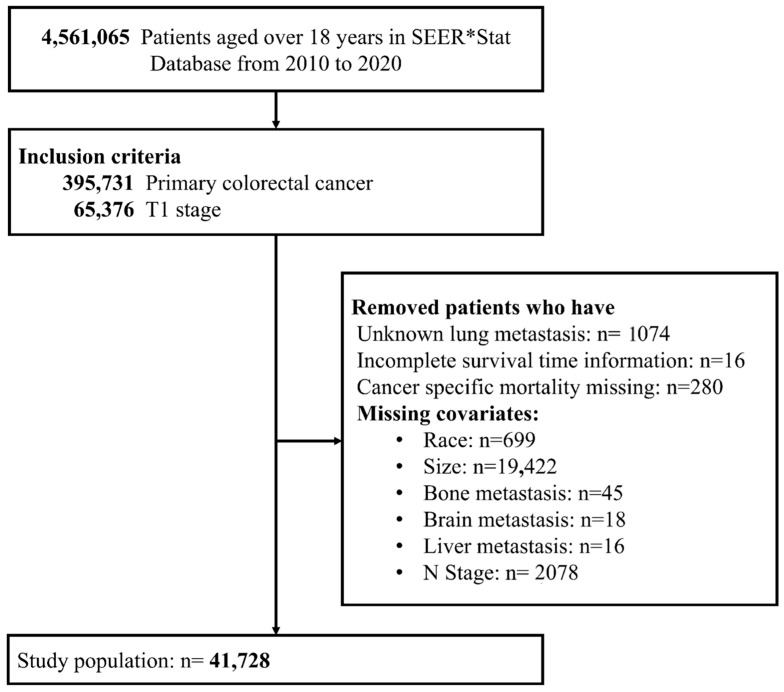
Flow diagram of the study population selection process. Percentages at each exclusion step are calculated from the 65,376 eligible patients. The largest single exclusion was missing tumor size (*n* = 19,422; 29.7%).

The study was initiated with 4,561,065 patients aged > 18 years from the SEER*Stat Database from 2010 to 2020. Based on the inclusion criteria, 65,376 patients with primary CRC and T1 stage were identified. Patients were excluded if they had unknown lung metastasis status (*n* = 1074), incomplete survival information (*n* = 16), missing information on cancer-specific mortality *(n* = 280), and missing study covariates including race/ethnicity (*n* = 699), tumor size (*n* = 19,422), bone metastasis status (*n* = 45), brain metastasis status (*n* = 18), liver metastasis status (*n* = 16), and N stage (*n* = 2078). Finally, 41,728 patients were included in the study (Figure 1).

The patients were divided into a training (*n* = 29,045) and validation (*n* = 12,683) cohort in a 7:3 ratio. Table 1 shows the demographic and clinical characteristics of the study population. The mean patient age was 64 years, and 52.2% were men. Most patients were non-Hispanic White (65.8%), with the primary tumor site located at the colon (65.6%), a tumor size ≤ 3 cm (81.4%), and N stage 0 (90.3%). The training and validation cohorts were balanced in terms of demographics and clinical status (all *p* > 0.05). Cancer-specific mortality was 11.3% and was higher in the training cohort (11.5%) than in the validation cohort (10.7%) (*p* = 0.016).

### 3.2. Characteristics of Patients with and Without Lung Metastasis

In the training cohort (Table 2), patients with lung metastasis had a significantly higher overall (89.7% vs. 23.2%, *p* < 0.001) and cancer-specific (85.3% vs. 10.2%, *p* < 0.001) mortality than those without lung metastasis.

Patients with lung metastasis were slightly older (65.0 years vs. 64.0 years, *p* = 0.036) and had a higher percentage of non-Hispanic black (15.6% vs. 11.9%), non-Hispanic American Indian/Alaska Native (1.2% vs. 0.7%), and Hispanic (14.7% vs. 12.2%) patients (*p* = 0.015). They also had a higher percentage of primary tumors in overlapping site of the colon/unspecified and rectosigmoid junction (9.5% vs. 6.0%, 35.2% vs. 27.2%, *p* < 0.001), tumor stage III (11.7% vs. 5.6%, *p* < 0.001), larger tumor sizes (56.2% vs. 13.8% for tumors between 3 and 6 cm and 23.2% vs. 3.6% for tumor size ≥ 6 cm, *p* < 0.001), and N stage 1–2 (63.8% vs. 90.7%, *p* < 0.001). Furthermore, patients with lung metastases exhibited a higher prevalence of bone (10.7% vs. 0.3%, *p* < 0.001), brain (1.2% vs. 0.1%, *p* < 0.001), and liver (74.3% vs. 3.6%, *p* < 0.001) metastases. Similar distribution was observed in the validation cohort (Supplementary Table S1).

### 3.3. Factors Associated with the Presence of Lung Metastasis

Univariate and multiple logistic regression analyses were performed to identify factors associated with the presence of lung metastasis. The results are summarized in Table 3. In the univariate analysis, significant factors associated with lung metastasis included age, race/ethnicity, primary site, and tumor characteristics such as grade, size, presence of other metastases, and N stage. The primary site, tumor grade, tumor size, metastasis to the bone, metastasis to the liver, and N stage were selected through the stepwise selection method using a statistical model.

**Figure 2:**
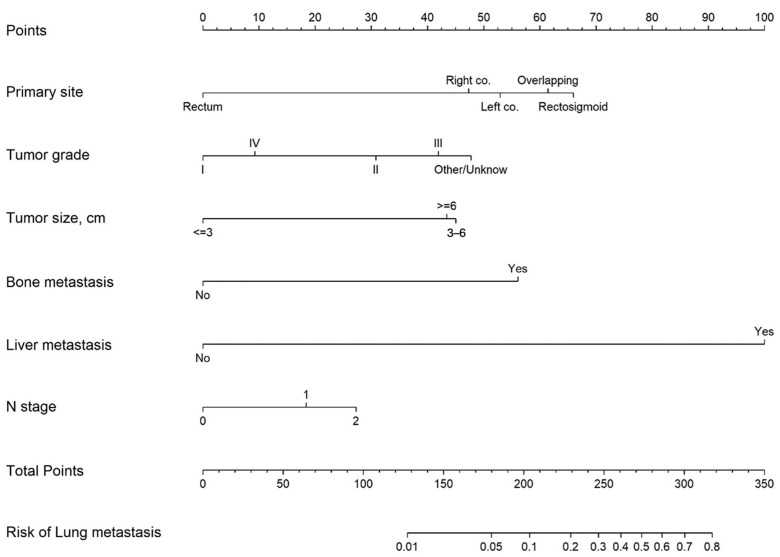
Nomogram for the presence of lung metastasis in the training cohort. Worked example: A 62-year-old patient with a grade II, 4.5 cm adenocarcinoma at the rectosigmoid junction, N1 disease, and no liver or bone metastasis would receive the following points: rectosigmoid junction = 65 points, grade II = 30 points, 4.5 cm = 45 points, N1 = 18 points, no bone metastasis = 0 points, and no liver metastasis = 0 points. The total of 158 points corresponds to an approximately 4% risk of developing lung metastasis.

As a result, primary tumors located at the “overlapping site of the colon/unspecified” (adjusted OR [aOR]: 1.56, 95% CI: 1.06–2.29, *p* = 0.024) and rectosigmoid junction (aOR: 1.80, 95% CI: 1.39–2.32, *p* < 0.001) were significantly associated with higher risks for lung metastasis as compared with tumors located at the right colon. Primary tumors located in the rectum were associated with a lower likelihood of lung metastasis (Table 3 and Figure 2).

A higher tumor grade was associated with an increased likelihood of lung metastasis compared to grade I (grades II, III, and other/unknown: aOR: 2.64, 3.75, and 4.50, all *p* < 0.001).

Larger tumors were significantly associated with lung metastasis. Specifically, tumors measuring 3–6 cm were associated with a four-fold increase in the likelihood of lung metastasis (aOR: 4.14; 95% CI: 3.16–5.41; *p* < 0.001), and tumors > 6 cm were nearly as strongly associated (aOR: 3.93; 95% CI: 2.82–5.47; *p* < 0.001).

The presence of liver metastasis was significantly and strongly predictive of lung metastasis (aOR: 23.36, 95% CI: 18.22–29.95, *p* < 0.001), followed by the presence of bone metastasis (aOR: 5.86, 95% CI: 3.73–9.19, *p* < 0.001).

Finally, as the N stage increased, the risk of lung metastasis also increased (N1 and 2: aOR = 1.79 and 2.36, both *p* < 0.001) (Table 3).

Of note, the rectum category contained only 264 patients in the training cohort, of whom only 2 had lung metastasis, resulting in a wide confidence interval (aOR 0.23, 95% CI 0.05–0.99) and borderline significance (*p* = 0.049). This estimate should be interpreted with caution, given the sparse-data instability. Rectum was retained as a separate category because of its biologically distinct venous drainage pattern (systemic vs. portal) with established relevance for metastatic patterns, but the estimate is imprecise.

### 3.4. Nomogram and ROC Curve Analysis

A nomogram was developed based on Model B (full post-staging model) to identify the factors associated with lung metastasis (Figure 2). Two models were evaluated: Model A (clinicopathologic-only: primary site, tumor grade, tumor size, N stage) and Model B (Model A variables plus liver and bone metastasis status).

**Figure 3:**
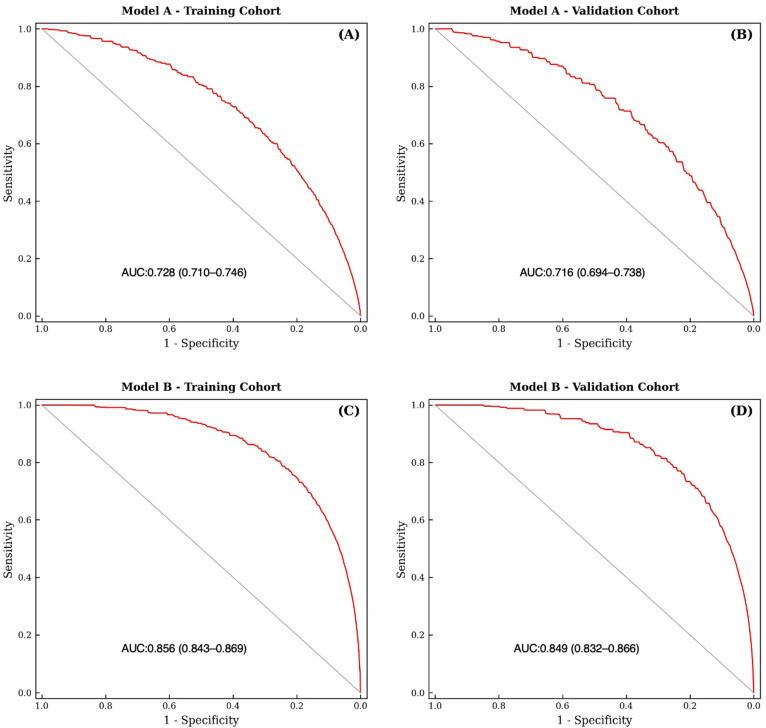
Receiver operating characteristic (ROC) curve analysis for synchronous lung metastasis prediction. (**A**) Model A (clinicopathologic-only)—training cohort; (**B**) Model A—validation cohort; (**C**) Model B (full post-staging model)—training cohort; (**D**) Model B—validation cohort. Model A is the primary model for pre-imaging risk stratification; Model B is the secondary post-staging descriptive model.

Model A demonstrated moderate discriminatory ability in the training cohort (AUC = 0.728, 95% CI: 0.710–0.746) and validation cohort (AUC = 0.716, 95% CI: 0.694–0.738). Model B demonstrated good discriminatory ability in the training cohort (AUC = 0.856, 95% CI: 0.843–0.869) and validation cohort (AUC = 0.849, 95% CI: 0.832–0.866) (Figure 3). The substantial increment in AUC from Model A to Model B (ΔAUC = 0.128) is primarily attributable to the inclusion of liver metastasis (aOR 23.36), which is present in 74.3% of patients with lung metastasis versus 3.6% without. This reflects shared metastatic biology rather than independent lung-specific prediction.

**Figure 4:**
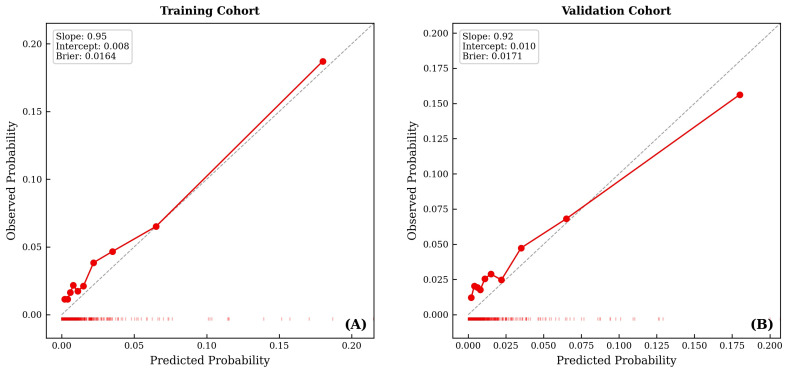
Calibration curves for Model A (clinicopathologic-only) nomogram-predicted probability of synchronous lung metastasis in the (**A**) training cohort (slope = 0.95, intercept = 0.008, Brier = 0.0164) and (**B**) validation cohort (slope = 0.92, intercept = 0.010, Brier = 0.0171).

Calibration curves for Model A indicated good agreement between predicted and observed probabilities (calibration slope 0.95, intercept 0.008, Brier score 0.0164) (Figure 4). Decision curve analysis demonstrated that Model A provides clinical net benefit over default strategies (treat all or treat none) within the threshold probability range of 1–8%. At a 2% threshold, the model implies pursuing chest CT for all patients with ≥2% predicted risk—a reasonable trade-off given that chest CT is a low-radiation, non-invasive study (approximately 49 patients imaged per one lung metastasis detected). At a 5% threshold, approximately 19 patients would be imaged per detection.

## 4. Discussion

Our study identified several significant predictors of sLM in patients with T1 CRC. The nomogram developed based on these predictors demonstrated that tumor grade, tumor size, primary tumor location, and lymph node involvement were the most influential factors in determining the risk of sLM. Specifically, patients with poorly differentiated tumors (grade III or IV), larger tumor sizes (≥3 cm), rectosigmoid junction or overlapping sites of colon involvement, and positive lymph nodes (N1 or N2) had a significantly higher risk of developing sLM compared to their counterparts. These findings underscore the importance of considering multiple clinicopathological factors when assessing the risk of distant metastasis, particularly in patients with early-stage CRC. Importantly, our analysis reveals two distinct analytic frameworks: Model A (clinicopathologic-only) addresses the clinically harder but more actionable question of predicting lung metastasis from variables available before staging imaging (AUC 0.728), while Model B (including concurrent organ metastases) addresses a descriptively easier question—identifying lung metastasis patterns when other metastatic sites are already documented (AUC 0.856). These two models serve fundamentally different clinical needs and should not be conflated.

The clinicopathologic model (Model A) demonstrated moderate discrimination (AUC 0.728), consistent with what is expected for predicting a rare endpoint (1.7% prevalence) using clinicopathologic variables alone. This performance is comparable to similar SEER-based nomogram studies for rare metastatic endpoints. The full post-staging model (Model B, AUC 0.856) demonstrated good discrimination, but this is largely attributable to the inclusion of concurrent organ metastasis variables—particularly liver metastasis—which reflects shared metastatic biology rather than independent lung-specific prediction. Our models are intended as complementary tools to existing staging approaches, not as replacements for TNM classification [19,20]. These results highlight the potential of the nomogram as a powerful tool for risk stratification and personalized decision-making in the management of patients with T1 CRC.

Tumor location has emerged as a significant predictor of sLM in patients with T1 CRC. Overlapping or unspecified tumor site was associated with a higher risk compared to tumors localized to a single segment of the colon or rectum (aOR 1.56, 95% CI 1.06–2.29, *p* = 0.024). Rectosigmoid junction tumors also had a higher risk compared to right colon tumors (aOR 1.80, 95% CI 1.39–2.32, *p* < 0.001). These findings suggest that tumors arising at different locations in the colorectum may have distinct biological characteristics that influence their metastatic potential. The stratification theory of left-sided colon cancer (LCC) and right-sided colon cancer (RCC) was proposed by American oncologist Bufill in 1990, highlighting the distinct molecular and genetic differences between the two types of CRC [21]. This theory has since been supported by numerous studies that have confirmed significant differences in the epidemiology, clinical presentation, comorbidities, and biological behaviors of LCC and RCC [22]. RCC, derived from the embryonic midgut, is associated with higher rates of BRAF mutations, CpG island hypermethylation, and microsatellite instability, whereas LCC, originating from the embryonic hindgut, more frequently exhibits chromosomal instability with mutations in KRAS, APC, SMAD4, and TP53 [23]. These molecular differences contribute to the distinct clinical outcomes observed, with RCC generally having a poorer prognosis and higher rates of metastasis to the peritoneum compared to LCC, which more commonly metastasizes to the lung and liver [24,25]. Tumor grade is another important risk factor for lung metastasis. Poorly differentiated or undifferentiated tumors (grade III) had a significantly higher risk compared with well-differentiated tumors (grade I) (aOR 3.75, 95% CI 2.02–6.94, *p* < 0.001), whereas moderately differentiated tumors (grade II) also had an increased risk (aOR 2.64, 95% CI 1.53–4.55, *p* < 0.001). These findings are consistent with those of previous studies that demonstrated the prognostic significance of tumor grade in CRC [7,8,26]. Poorly differentiated tumors are characterized by a higher degree of cellular atypia, loss of glandular architecture, and increased proliferative activity, which may confer a greater ability for cancer cells to invade, migrate, and establish distant metastases [7,27]. Specifically, poorly differentiated colorectal adenocarcinomas can be classified into two subtypes: Por1, which is characterized by solid growth with little stroma, and Por2, which is characterized by a trabecular structure rich in fibrous stroma. The Por2 subtype is associated with a higher frequency of metastasis to the lungs and other organs, and a poorer survival rate than Por1 [28,29]. The molecular pathways underlying the link between tumor differentiation and metastatic potential warrant further investigation.

Tumor size is also a significant predictor of lung metastasis, with larger tumors being associated with a higher risk. Tumors measuring 3–6 cm (aOR 4.14, 95% CI 3.16–5.41, *p* < 0.001) and those ≥6 cm (aOR 3.93, 95% CI 2.82–5.47, *p* < 0.001) had a significantly increased risk compared to tumors ≤ 3 cm. This finding is in line with previous studies that have reported an association between larger tumor size and worse outcomes in CRC [8,30,31]. Further supporting this, research on pulmonary metastasectomy for CRC indicated that larger pulmonary metastases were linked to poorer survival outcomes, emphasizing the prognostic importance of tumor size [32]. Larger tumors may have a greater propensity for metastatic spread owing to increased invasiveness, angiogenesis, or the presence of occult metastases not detectable by current imaging modalities [33,34].

The presence of lymph node metastases was a strong predictor of lung metastasis, and N1 (aOR 1.79, 95% CI 1.39–2.28, *p* < 0.001) and N2 (aOR 2.36, 95% CI 1.45–3.86, *p* < 0.001) stage tumors had a higher risk compared to N0 tumors. This finding underscores the importance of lymph node involvement as a marker of more advanced disease and its role in the metastatic cascade. Lymph node metastasis is a critical step in the progression of CRC, reflecting the ability of cancer cells to invade lymphatic vessels and establish growth in regional lymph nodes [35,36].

The presence of concurrent metastases in other organs, such as the liver (aOR: 23.36, 95% CI: 18.22–29.95, *p* < 0.001), bone (aOR: 5.86, 95% CI: 3.73–9.19, *p* < 0.001), or brain (univariate OR: 21.89, 95% CI: 8.53–56.17, *p* < 0.001), was strongly associated with an increased risk of lung metastasis. This finding suggests that the primary tumor has already developed molecular machinery for metastatic spread and that the presence of metastases in one organ may increase the likelihood of metastases in other organs [7,37,38,39]. The concept of "organotropism" in metastatic spread, where cancer cells have a propensity to metastasize to specific organ sites, has been increasingly recognized [40,41]. The molecular determinants that guide CRC cells to specific organs, such as the liver and lungs, warrant further investigation. Understanding the biological mechanisms underlying organotropism may help in the development of targeted therapies to prevent or treat metastatic diseases.

One of the major strengths of this study was the large sample size and population-based nature of the SEER database, which enhanced the generalizability and robustness of the findings. The SEER database covers approximately 28% of the U.S. population and includes diverse geographic regions and demographic groups [42,43]. This wide coverage ensures that our results are representative of the general population and reduces the risk of selection bias inherent in single-center or regional studies [44]. Furthermore, the large sample size provided sufficient statistical power to detect significant associations and develop a reliable nomogram [45].

Another key strength of our study is the comprehensive set of clinicopathological variables incorporated into the nomogram, which provides a more holistic approach to risk assessment than existing models. Although previous studies have identified individual factors associated with sLM in CRC, such as tumor location, grade, and lymph node status [10,46], few studies have integrated these variables into a single predictive model. Our nomogram offers a more comprehensive and accurate risk assessment tool for patients with T1 CRC by including a wide range of readily available clinical and pathological factors [47].

We used a split-sample approach to develop and validate the nomogram, with 70% of the data used for training and the remaining 30% for validation at random. This approach helps to prevent overfitting and provides a more realistic assessment of a nomogram’s performance in independent datasets [48]. Furthermore, we evaluated the discriminatory ability of the nomogram using the AUC and assessed its calibration using calibration plots [49]. These statistical measures provide a comprehensive evaluation of the performance of a nomogram and enhance the credibility of the findings. Future translation of such nomograms into clinical decision support tools should follow rigorous validation frameworks, as exemplified by the CLASSICA software-as-a-medical-device trial protocol [50].

Several limitations must be acknowledged. First, and most importantly, complete-case analysis excluded 31.7% of eligible patients primarily due to missing tumor size data. Excluded patients had similar demographic distributions (age, sex, race; all *p* > 0.05) but higher distant metastasis rates (2.5% vs. 1.7%, *p* < 0.001) and were disproportionately from earlier diagnosis years (Supplementary Table S3). This suggests potential selection bias, and our results are most applicable to patients with a complete staging workup. Future studies should employ multiple imputation or sensitivity analyses under different missing-data assumptions to bound potential bias.

Second, the inclusion of concurrent organ metastasis variables (liver, bone) in Model B constitutes predictor leakage: these variables share the underlying process of metastatic dissemination with the outcome (lung metastasis), which inflates apparent discrimination. The substantial AUC drop from Model B (0.856) to Model A (0.728) confirms this concern. Model A provides a more honest estimate of clinicopathologic prediction performance, while Model B should be interpreted as a descriptive post-staging tool rather than a truly predictive instrument.

Third, we used split-sample validation, which is less rigorous than bootstrap optimism-correction. While our calibration metrics (slope 0.95, Brier score 0.0164) are reassuring, optimism-corrected estimates are recommended for future analyses. External validation in independent cohorts—such as the National Cancer Database, European cancer registries, or a SEER temporal split—is essential before clinical adoption.

Fourth, our stepwise variable selection approach (entry *p* < 0.5, retention *p* < 0.05) may introduce selection instability, though backward elimination with *p* < 0.05 confirmed the same variable set. Penalized regression methods (e.g., LASSO) would be preferable for future work.

Fifth, the association of “unknown” tumor grade with lung metastasis risk may reflect incomplete diagnostic evaluation rather than true biological differences. Sixth, the rectum category (*n* = 264, 2 events) provides an imprecise estimate due to sparse data. Finally, the SEER database covers approximately 28% of the US population, and the retrospective design and lack of molecular biomarker data (e.g., circulating tumor DNA, CEA levels, microsatellite instability status) limit predictive accuracy. Future research should focus on prospective validation, integration of molecular markers, and assessment of the nomogram’s impact on patient outcomes.

## 5. Conclusions

In this study, a nomogram was developed and validated using the SEER database to predict sLM in patients with T1 CRC. The nomogram identified key clinicopathological variables such as tumor location, grade, size, and lymph node status to provide personalized risk assessments. Significant predictors of sLM included poorly differentiated tumors, larger tumor sizes, rectosigmoid junctions or overlapping colon involvement, positive lymph nodes, and concurrent metastases in other organs. Model A (clinicopathologic-only) demonstrated moderate discrimination (AUC 0.728), suitable for pre-imaging risk stratification, while Model B (post-staging) demonstrated good discrimination (AUC 0.856) for comprehensive risk profiling. Both models showed acceptable calibration. These nomograms may serve as adjunct tools for risk-stratified clinical decision-making in T1 CRC, pending external validation.

## Figures and Tables

**Table 1:** Characteristics of the patients with T1 CRC.

Characteristics	Total (*n* = 41,728)	Cohort		*p*-Value
		Training (*n* = 29,045)	Validation (*n* = 12,683)	
**Outcome**				
Overall mortality	10,129 (24.3)	7077 (24.4)	3052 (24.1)	0.508
Cancer-specific mortality	4696 (11.3)	3340 (11.5)	1356 (10.7)	**0.016**
**Demography**				
Age, years	64 ± 14.1	65 ± 13.2	64 ± 14.1	0.926
18–29	795 (1.9)	568 (2.0)	227 (1.8)	0.445
30–39	1231 (3.0)	878 (3.0)	353 (2.8)	
40–49	3092 (7.4)	2133 (7.3)	959 (7.6)	
50–59	10,096 (24.2)	6969 (24.0)	3127 (24.7)	
60–69	11,214 (26.9)	7808 (26.9)	3406 (26.9)	
70–79	9333 (22.4)	6532 (22.5)	2801 (22.1)	
80+	5967 (14.3)	4157 (14.3)	1810 (14.3)	
Sex				0.655
Male	21,777 (52.2)	15,137 (52.1)	6640 (52.4)	
Female	19,951 (47.8)	13,908 (47.9)	6043 (47.6)	
Race/ethnicity				0.531
Non-Hispanic White	27,447 (65.8)	19,062 (65.6)	8385 (66.1)	
Non-Hispanic Black	4929 (11.8)	3476 (12.0)	1453 (11.5)	
Non-Hispanic API	3946 (9.5)	2739 (9.4)	1207 (9.5)	
Non-Hispanic AI/AN	326 (0.8)	220 (0.8)	106 (0.8)	
Hispanic	5080 (12.2)	3548 (12.2)	1532 (12.1)	
**Clinical status**				
Primary site				0.397
Right colon	16,478 (39.5)	11,540 (39.7)	4938 (38.9)	
Left colon	10,919 (26.2)	7531 (25.9)	3388 (26.7)	
Overlapping site of the colon/Unspecified	2516 (6.0)	1762 (6.1)	754 (5.9)	
Rectosigmoid junction	11,442 (27.4)	7948 (27.4)	3494 (27.5)	
Rectum	373 (0.9)	264 (0.9)	109 (0.9)	
Tumor grade				0.708
I	10,502 (25.2)	7306 (25.2)	3196 (25.2)	
II	22,270 (53.4)	15,453 (53.2)	6817 (53.7)	
III	2358 (5.7)	1664 (5.7)	694 (5.5)	
IV	334 (0.8)	234 (0.8)	100 (0.8)	
Other/Unknown	6264 (15.0)	4388 (15.1)	1876 (14.8)	
Tumor size, cm				0.681
≤3	33,981 (81.4)	23,678 (81.5)	10,303 (81.2)	
3 < Size < 6	6097 (14.6)	4215 (14.5)	1882 (14.8)	
≥6	1650 (4.0)	1152 (4.0)	498 (3.9)	
Organ metastasis				
Bone metastasis	185 (0.4)	132 (0.5)	53 (0.4)	0.605
Brain metastasis	35 (0.1)	22 (0.1)	13 (0.1)	0.385
Liver metastasis	1963 (4.7)	1395 (4.8)	568 (4.5)	0.150
Lung metastasis	706 (1.7)	495 (1.7)	211 (1.7)	0.767
N stage				0.489
0	37,681 (90.3)	26,195 (90.2)	11,486 (90.6)	
1	3561 (8.5)	2509 (8.6)	1052 (8.3)	
2	486 (1.2)	341 (1.2)	145 (1.1)	

API, Asian/Pacific islander; AI/AN, American Indian/Alaska Native; CRC, colorectal cancer. Significant results are shown in bold.

**Table 2:** Characteristics of the patients with T1 CRC, with and without the presence of lung metastasis, in the training cohort.

Characteristics	Total (*n* = 29,045)	Lung Metastasis		*p*-Value
		with (*n* = 495)	Without (*n* = 28,550)	
**Outcome**				
Overall mortality	7077 (24.4)	444 (89.7)	6633 (23.2)	**<0.001**
Cancer-specific mortality	3340 (11.5)	422 (85.3)	2918 (10.2)	**<0.001**
**Demography**				
Age, years	64.0 ± 14.1	65.0 ± 13.2	64.0 ± 14.1	**0.036**
18–29	568 (2.0)	1 (0.2)	567 (2.0)	**0.008**
30–39	878 (3.0)	12 (2.4)	866 (3.0)	
40–49	2133 (7.3)	52 (10.5)	2081 (7.3)	
50–59	6969 (24.0)	117 (23.6)	6852 (24.0)	
60–69	7808 (26.9)	127 (25.7)	7681 (26.9)	
70–79	6532 (22.5)	105 (21.2)	6427 (22.5)	
80+	4157 (14.3)	81 (16.4)	4076 (14.3)	
Sex				0.648
Male	15,137 (52.1)	263 (53.1)	14,874 (52.1)	
Female	13,908 (47.9)	232 (46.9)	13,676 (47.9)	
Race/ethnicity				**0.015**
Non-Hispanic White	19,062 (65.6)	298 (60.2)	18,764 (65.7)	
Non-Hispanic Black	3476 (12.0)	77 (15.6)	3399 (11.9)	
Non-Hispanic API	2739 (9.4)	41 (8.3)	2698 (9.5)	
Non-Hispanic AI/AN	220 (0.8)	6 (1.2)	214 (0.7)	
Hispanic	3548 (12.2)	73 (14.7)	3475 (12.2)	
**Clinical status**				
Primary site				**<0.001**
Right colon	11,540 (39.7)	154 (31.1)	11,386 (39.9)	
Left colon	7531 (25.9)	118 (23.8)	7413 (26.0)	
Overlapping site of the colon/Unspecified	1762 (6.1)	47 (9.5)	1715 (6.0)	
Rectosigmoid junction	7948 (27.4)	174 (35.2)	7774 (27.2)	
Rectum	264 (0.9)	2 (0.4)	262 (0.9)	
Tumor grade				**<0.001**
I	7306 (25.2)	15 (3.0)	7291 (25.5)	
II	15,453 (53.2)	199 (40.2)	15,254 (53.4)	
III	1664 (5.7)	58 (11.7)	1606 (5.6)	
IV	234 (0.8)	4 (0.8)	230 (0.8)	
Other/Unknown	4388 (15.1)	219 (44.2)	4169 (14.6)	
Tumor size, cm				**<0.001**
≤3	23,678 (81.5)	102 (20.6)	23,576 (82.6)	
3 < Size < 6	4215 (14.5)	278 (56.2)	3937 (13.8)	
≥6	1152 (4.0)	115 (23.2)	1037 (3.6)	
Organ metastasis				
Bone metastasis	132 (0.5)	53 (10.7)	79 (0.3)	**<0.001**
Brain metastasis	22 (0.1)	6 (1.2)	16 (0.1)	**<0.001**
Liver metastasis	1395 (4.8)	368 (74.3)	1027 (3.6)	**<0.001**
N stage				**<0.001**
0	26,195 (90.2)	316 (63.8)	25,879 (90.6)	
1	2509 (8.6)	150 (30.3)	2359 (8.3)	
2	341 (1.2)	29 (5.9)	312 (1.1)	

API, Asian/Pacific islander; AI/AN, American Indian/Alaska Native; CRC, colorectal cancer. Significant results are shown in bold.

**Table 3:** Factors associated with the presence of lung metastasis in the training cohort.

Variables	OR (95% CI)	*p* Value	aOR (95% CI)	*p* Value
Age (vs. 18–29 y)				NI
30–39	7.86 (1.02–60.58)	**0.048**		
40–49	14.17 (1.95–102.69)	**0.009**		
50–59	9.68 (1.35–69.43)	**0.024**		
60–69	9.37 (1.31–67.18)	**0.026**		
70–79	9.26 (1.29–66.49)	**0.027**		
80+	11.27 (1.57–81.11)	**0.016**		
Sex (vs. Female)				NI
Male	1.04 (0.87–1.25)	0.649		
Race/ethnicity (vs. Non-Hispanic white)				NI
Non-Hispanic black	1.43 (1.11–1.84)	**0.006**		
Non-Hispanic API	0.96 (0.69–1.33)	0.793		
Non-Hispanic AI/AN	1.77 (0.78–4.01)	0.174		
Hispanic	1.32 (1.02–1.71)	**0.034**		
Primary site (vs. Right colon)				
Left colon	1.18 (0.92–1.50)	0.186	1.19 (0.9–1.57)	0.212
Overlapping sites of the colon/Unspecified	2.03 (1.46–2.82)	**<0.001**	1.56 (1.06–2.29)	**0.024**
Rectosigmoid junction	1.65 (1.33–2.06)	**<0.001**	1.80 (1.39–2.32)	**<0.001**
Rectum	0.56 (0.14–2.29)	0.424	0.23 (0.05–0.99)	**0.049**
Tumor grade (vs. I)				
II	6.34 (3.75–10.72)	**<0.001**	2.64 (1.53–4.55)	**<0.001**
III	17.55 (9.92–31.05)	**<0.001**	3.75 (2.02–6.94)	**<0.001**
IV	8.45 (2.78–25.67)	**<0.001**	1.34 (0.39–4.58)	0.643
Other/Unknown	25.53 (15.11–43.14)	**<0.001**	4.50 (2.59–7.81)	**<0.001**
Tumor size (vs. ≤3 cm)				
3–6	16.32 (12.98–20.53)	**<0.001**	4.14 (3.16–5.41)	**<0.001**
≥6	25.63 (19.49–33.7)	**<0.001**	3.93 (2.82–5.47)	**<0.001**
Organ metastasis				
Bone metastasis	43.24 (30.15–62)	**<0.001**	5.86 (3.73–9.19)	**<0.001**
Brain metastasis	21.89 (8.53–56.17)	**<0.001**		
Liver metastasis	77.65 (62.87–95.9)	**<0.001**	23.36 (18.22–29.95)	**<0.001**
N stage (vs. 0)				
1	5.21 (4.27–6.35)	**<0.001**	1.79 (1.39–2.28)	**<0.001**
2	7.62 (5.13–11.32)	**<0.001**	2.36 (1.45–3.86)	**<0.001**

NI, Not included in multivariate stepwise Logistic regression models; OR, odds ratio; CI, confidence interval; API, Asian/Pacific islander; AI/AN, American Indian/Alaska native. Significant results are shown in bold.

## Data Availability

The data used in this study are publicly available from the Surveillance, Epidemiology, and End Results (SEER) database of the National Cancer Institute (https://seer.cancer.gov/). Access to the data is subject to SEER data use agreements.

## Supplementary Materials

The following supporting information can be downloaded at https://www.mdpi.com/article/10.3390/medicina62030431/s1. Table S1: Pattern of Missing Data in Study Cohort, Table S2: Characteristics of the patients with T1 CRC, with and without lung metastasis, in the validation cohort., Table S3: Comparison of Included vs. Excluded Patients, Table S4: Model Performance With and Without Other Organ Metastasis Variables, Table S5: Model Performance at Different Risk Thresholds, Table S6: Variable Definitions and SEER Codes.

## Abbreviations

The following abbreviations are used in this manuscript:

AI/AN	American Indian/Alaska Native
API	Asian/Pacific Islander
AUC	Area Under the Curve
CI	Confidence Interval
CRC	Colorectal Cancer
EMR	Electronic Medical Records
LCC	Left-Sided Colon Cancer
NCI	National Cancer Institute
OR	Odds Ratio
aOR	Adjusted Odds Ratio
POR1	Poorly Differentiated Adenocarcinoma, Solid Type
POR2	Poorly Differentiated Adenocarcinoma, Non-Solid (Trabecular) Type
RCC	Right-Sided Colon Cancer
ROC	Receiver Operating Characteristic
SEER	Surveillance, Epidemiology, and End Results
sLM	Synchronous Lung Metastasis
