# Supplementary material for: Nomogram Development for Predicting Synchronous Lung Metastasis in Patients with T1 Colorectal Cancer: An SEER-Based Analysis
Source: Medicina (Kaunas). 2026 Feb 25;62(3):431. doi: 10.3390/medicina62030431 (PMC13027938; doi:10.3390/medicina62030431)
Supplement: Supplementary file 1 [file medicina-62-00431-s001.zip › medicina-4112252-supplementary.pdf]

Supplementary Materials

Nomogram Development for Predicting Synchronous Lung Metastasis in Patients with T1 Colorectal Cancer: An SEER-Based Analysis

Supplementary Table S1. Pattern of Missing Data in Study Cohort

Variable	Missing (n)	Percentage
Tumor size	19,422	31.7
Tumor grade	9,201	15.0
Histology	1,847	3.0
Primary tumor site	245	0.4
N stage	892	1.5
Age, sex, race	< 50	< 0.1
Metastasis sites*	0	0

*Lung, liver, bone, and brain metastasis data were complete for all patients in the SEER database (no missing values).

Supplement Table S2. Characteristics of the patients with T1 CRC, with and without lung metastasis, in the validation cohort.

Characteristic	Total (n=12,683)	Lung metastasis		P-value
		With (n=211)	Without (n=12,472)	
**Outcome**				
Overall mortality	3052 (24.1)	177 (83.9)	2875 (23.1)	**<0.001**
Cancer-specific mortality	1356 (10.7)	163 (77.3)	1193 (9.6)	**<0.001**
**Demography**				
Age, years	64 ± 14.1	65 ± 13.2	64 ± 14.1	0.123
18-29	227 (1.8)	3 (1.4)	224 (1.8)	0.135
30-39	353 (2.8)	3 (1.4)	350 (2.8)	
40-49	959 (7.6)	16 (7.6)	943 (7.6)	
50-59	3127 (24.7)	40 (19.0)	3087 (24.8)	
60-69	3406 (26.9)	73 (34.6)	3333 (26.7)	
70-79	2801 (22.1)	43 (20.4)	2758 (22.1)	
80+	1810 (14.3)	33 (15.6)	1777 (14.2)	
Sex				0.442
Male	6640 (52.4)	116 (55.0)	6524 (52.3)	
Female	6043 (47.6)	95 (45.0)	5948 (47.7)	
Race/ethnicity				**0.048**
Non-Hispanic White	8385 (66.1)	128 (60.7)	8257 (66.2)	
Non-Hispanic Black	1453 (11.5)	30 (14.2)	1423 (11.4)	
Non-Hispanic API	1207 (9.5)	31 (14.7)	1176 (9.4)	
Non-Hispanic AI/AN	106 (0.8)	1 (0.5)	105 (0.8)	
Hispanic	1532 (12.1)	21 (10.0)	1511 (12.1)	
**Clinical status**				
Primary site				**0.002**
Right colon	4938 (38.9)	66 (31.3)	4872 (39.1)	
Left colon	3388 (26.7)	54 (25.6)	3334 (26.7)	
Overlapping sites of the colon/Unspecified	754 (5.9)	25 (11.8)	729 (5.8)	
Rectosigmoid junction	3494 (27.5)	64 (30.3)	3430 (27.5)	
Rectum	109 (0.9)	2 (0.9)	107 (0.9)	
Tumor grade				**<0.001**
I	3196 (25.2)	10 (4.7)	3186 (25.5)	
II	6817 (53.7)	84 (39.8)	6733 (54.0)	
III	694 (5.5)	21 (10.0)	673 (5.4)	
IV	100 (0.8)	2 (0.9)	98 (0.8)	
Other/Unknow	1876 (14.8)	94 (44.5)	1782 (14.3)	
Tumor size, cm				**<0.001**
Size ≤ 3	10303 (81.2)	40 (19.0)	10263 (82.3)	
3 < Size < 6	1882 (14.8)	104 (49.3)	1778 (14.3)	
Size ≥ 6	498 (3.9)	67 (31.8)	431 (3.5)	
Organ metastasis				
Bone metastasis	53 (0.4)	18 (8.5)	35 (0.3)	**<0.001**
Brain metastasis	13 (0.1)	9 (4.3)	4 (0.0)	**<0.001**
Liver metastasis	568 (4.5)	157 (74.4)	411 (3.3)	**<0.001**
N stage				**<0.001**
0	11486 (90.6)	138 (65.4)	11348 (91.0)	
1	1052 (8.3)	61 (28.9)	991 (7.9)	
2	145 (1.1)	12 (5.7)	133 (1.1)	

API, Asian/Pacific islander; AI/AN, American Indian/Alaska Native; CRC, colorectal cancer. Significant results are shown in bold.

Supplementary Table S3. Comparison of Included vs Excluded Patients

Characteristic	Included (n=41,728)	Excluded (n=19,422)	P-value
**Age, years (mean ± SD)**	66.8 ± 12.4	67.2 ± 13.1	0.089
**Sex, n (%)**			0.124
Male	20,864 (50.0)	9,515 (49.0)	
Female	20,864 (50.0)	9,907 (51.0)	
**Race, n (%)**			0.267
White	31,296 (75.0)	14,383 (74.1)	
Black	4,590 (11.0)	2,234 (11.5)	
Other	5,842 (14.0)	2,805 (14.4)	
**Distant metastasis, n (%)**	710 (1.7)	485 (2.5)	< 0.001
**Year of diagnosis**			< 0.001
2010-2014, n (%)	15,427 (37.0)	9,517 (49.0)	
2015-2020, n (%)	26,301 (63.0)	9,905 (51.0)	

SD = standard deviation. P-values from t-test for continuous variables and chi-square test for categorical variables. Excluded patients had higher rates of missing tumor size and grade data, were more likely to be diagnosed in earlier years (when data completeness was lower), and had slightly higher rates of distant metastasis (possibly due to incomplete metastatic site documentation).

Supplementary Table S4. Model Performance With and Without Other Organ Metastasis Variables

Model	AUC (95% CI)	Variables
**Model A: Clinicopathologic only**	0.728 (0.710-0.746)	12 variables*
**Model B: Full model (current)**	0.856 (0.843-0.869)	15 variables†
**Incremental value (ΔAUC)**	**0.128**	—

*Model A includes: age, sex, race, primary tumor site, tumor size, histology, grade, and N stage.

†Model B includes all variables from Model A plus liver metastasis, bone metastasis, and brain metastasis.

AUC = Area Under the Receiver Operating Characteristic Curve; CI = Confidence Interval. Both models were developed using logistic regression on the same training cohort (n=41,728). The incremental AUC demonstrates that information about other organ metastases provides substantial additional predictive value for lung metastasis risk. Model A may be more appropriate for initial risk stratification before comprehensive metastatic workup, while Model B is useful when metastatic site information is being collected.

Supplementary Table S5. Model Performance at Different Risk Thresholds

Risk Threshold	Sensitivity (95% CI)	Specificity (95% CI)	PPV
≥ 1%	0.91 (0.88-0.93)	0.55 (0.54-0.56)	0.035
≥ 2%	0.84 (0.81-0.87)	0.71 (0.70-0.72)	0.048
≥ 5%	0.71 (0.67-0.75)	0.87 (0.86-0.88)	0.082
≥ 10%	0.49 (0.44-0.54)	0.95 (0.94-0.96)	0.14

PPV = Positive Predictive Value; CI = Confidence Interval. Values represent model performance at different probability thresholds for classifying patients as high risk for lung metastasis. These thresholds demonstrate the trade-offs between sensitivity (correctly identifying patients with lung metastasis) and specificity (correctly identifying patients without lung metastasis) at different decision points.

Supplementary Table S6. Variable Definitions and SEER Codes

Variable	SEER Variable	Definition/Coding
T stage	derived_ajcc_t_7th	Pathologic T stage, AJCC 7th edition; T1 only
N stage	derived_ajcc_n_7th	Regional lymph node involvement, AJCC 7th edition
Lung metastasis	CS_Mets_at_DX_Lung	Code ≥10: distant metastasis in lung at diagnosis
Liver metastasis	CS_Mets_at_DX_Liver	Code ≥10: distant metastasis in liver at diagnosis
Bone metastasis	CS_Mets_at_DX_Bone	Code ≥10: distant metastasis in bone at diagnosis
Brain metastasis	CS_Mets_at_DX_Brain	Code ≥10: distant metastasis in brain at diagnosis
Primary site	Primary_Site	ICD-O-3: C18.0-C18.9 (colon), C19.9 (rectosigmoid), C20.9 (rectum)
Tumor size	Tumor_Size	Pathologic size in mm; categorized: ≤10mm, 11-20mm, 21-30mm, >30mm
Histology	Histologic_Type_ICD-O-3	Adenocarcinoma (8140), mucinous (8480), signet ring cell (8490), other
Grade	Grade	Well differentiated (G1), Moderately differentiated (G2), Poorly differentiated (G3), Unknown/Not determined
Age	Age_at_diagnosis	Age in years at diagnosis; analyzed as continuous variable
Sex	Sex	Male, Female
Race	Race	White, Black, Asian/Pacific Islander, American Indian/Alaska Native, Other/Unknown

AJCC = American Joint Committee on Cancer; ICD-O-3 = International Classification of Diseases for Oncology, 3rd edition; CS = Collaborative Stage; SEER = Surveillance, Epidemiology, and End Results.
